# Supplementary material for: Causal Relationship between Aging and Anorexia Nervosa: A White-Matter-Microstructure-Mediated Mendelian Randomization Analysis
Source: Biomedicines. 2024 Aug 16;12(8):1874. doi: 10.3390/biomedicines12081874 (PMC11351342; doi:10.3390/biomedicines12081874)
Supplement: Supplementary file 1 [file biomedicines-12-01874-s001.zip › Supplementary Figure/Figure S9/Leaveoneout plot of MR analysis for ubm-b-1655 to AN.pdf]

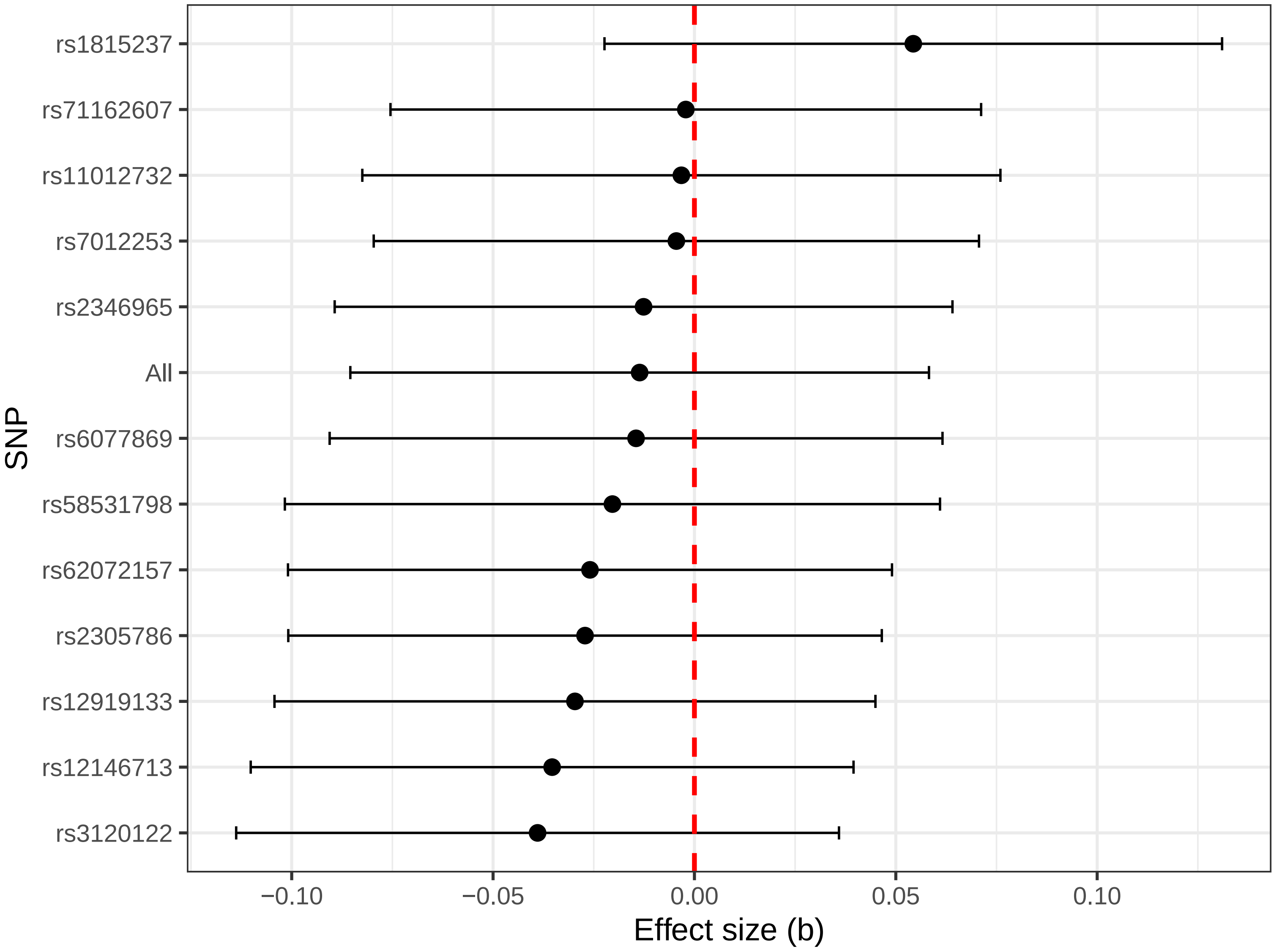
SNP
rs1815237
rs71162607
rs11012732
rs7012253
rs2346965
All
rs6077869
rs58531798
rs62072157
rs2305786
rs12919133
rs12146713
rs3120122
−0.10
−0.05
0.00
0.05
0.10
Effect size (b)
